# Effects of Chronic Inhalation of Electronic Cigarette Vapor Containing Nicotine on Neurobehaviors and Pre/Postsynaptic Neuron Markers

**DOI:** 10.3390/toxics10060338

**Published:** 2022-06-20

**Authors:** Fawaz Alasmari, Farraj M. Alotibi, Faleh Alqahtani, Tahani K. Alshammari, Aban A. Kadi, Abdullah M. Alghamdi, Bassil S. Allahem, Abdullah F. Alasmari, Shakir D. Alsharari, Salim S. Al-Rejaie, Musaad A. Alshammari

**Affiliations:** Department of Pharmacology and Toxicology, College of Pharmacy, King Saud University, Riyadh 11451, Saudi Arabia; 439105918@student.ksu.edu.sa (F.M.A.); afaleh@ksu.edu.sa (F.A.); talshammary@ksu.edu.sa (T.K.A.); 437103561@student.ksu.edu.sa (A.A.K.); 437101495@student.ksu.edu.sa (A.M.A.); 437100583@student.ksu.edu.sa (B.S.A.); afalasmari@ksu.edu.sa (A.F.A.); sdalsharari@ksu.edu.sa (S.D.A.); rejaie@ksu.edu.sa (S.S.A.-R.)

**Keywords:** e-cigarettes, nicotine dependence, neurobehavior, VGLUT1, VGAT, cytokine markers

## Abstract

Nicotine-exposed animal models exhibit neurobehavioral changes linked to impaired synaptic plasticity. Previous studies highlighted alterations in neurotransmitter levels following nicotine exposure. Vesicular glutamate transporter (VGLUT1) and vesicular gamma-aminobutyric acid (GABA) transporter (VGAT) are essential for the transport and release of glutamate and GABA, respectively, from presynaptic neurons into synapses. In our work, an e-cigarette device was used to deliver vapor containing nicotine to C57BL/6J mice for four weeks. Novel object recognition, locomotion, and Y-maze tests were performed to investigate the behavioral parameters. Protein studies were conducted to study the hippocampal expression of VGLUT1, VGAT, and postsynaptic density protein 95 (PSD95) as well as brain cytokine markers. Long-term memory and locomotion tests revealed that e-cigarette aerosols containing nicotine modulated recognition memory and motor behaviors. We found that vapor exposure increased VGLUT1 expression and decreased VGAT expression in the hippocampus. No alterations were found in PSD95 expression. We observed that vapor-containing nicotine exposure altered certain brain cytokines such as IFNβ-1 and MCP-5. Our work provides evidence of an association between neurobehavioral changes and altered hippocampal VGLUT1 and VGAT expression in mice exposed to e-cigarette vapors containing nicotine. Such exposure was also associated with altered neurobehaviors, which might affect neurodegenerative diseases.

## 1. Introduction

**Figure 1:**
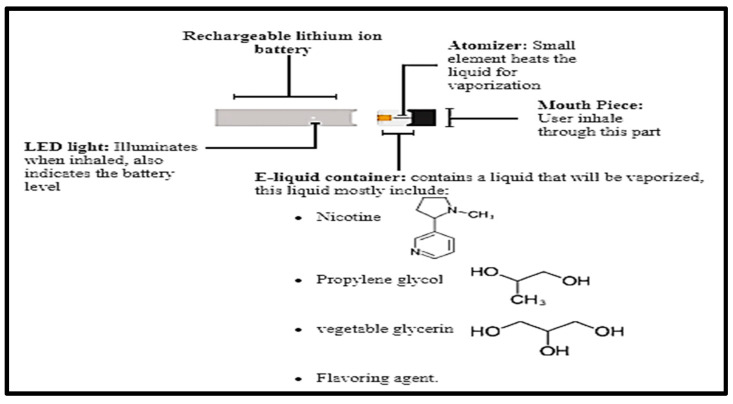
Electronic cigarette components.

Cigarette use is the largest predictable cause of numerous fatal diseases and increased mortality [1,2,3,4]. Electronic cigarettes (e-cigarettes) have been introduced as a safer and more convenient alternative to conventional cigarettes [5,6]. These operate by heating fluids to produce aerosols composed of compounds such as propylene glycol, aldehydes, glycerin, nicotine, and flavorings (Figure 1). However, studies have demonstrated that e-cigarettes are associated with toxicological effects that constitute major safety concerns [3,7,8,9,10,11]. Moreover, Etter and Eissenberg [12] noted that nicotine dependence might occur following e-cigarette consumption. A prospective study undertaken by Al-Delaimy, et al. [13] found that, compared with conventional smokers who never used e-cigarettes, individuals who experimented with the use of e-cigarettes were less likely to abstain from consumption of cigarettes. By contrast, Carpenter, et al. [14] reported that e-cigarette use was associated with positive changes in cessation-related behavior in adults.

Previous studies suggest that cigarette users are at an increased risk of altered cognitive performance characterized by changes in working memory, and motor functions [15,16]. Within the brain, the hippocampus is primarily associated with neurobehavioral functions such as learning and memory [17,18]; it is responsible for assembling information and facilitating the formation of complex representations, which are a key feature of long-term declarative memory and neurobehavioral changes [17,19]. However, the hippocampus is also characterized by a high level of synaptic plasticity, which considerably influences its function [20] and renders it susceptible to the effects of drugs such as nicotine, which could have profound effects on learning and memory-related behavioral changes [17,21]. These behavioral parameters are linked to neurodegenerative diseases such as Alzheimer’s disease [22]. The mechanisms by which nicotine influences the hippocampal region include activation of nicotinic acetylcholine receptors (nAChRs) [21,23]. 

Glutamate, the principal excitatory neurotransmitter in the human brain, is responsible for cognitive function, cortical excitability, and energy production [24,25,26]. E-cigarette vapor containing nicotine may increase glutamate transmission and modulate postsynaptic glutamate receptors [27,28]. Specifically, nicotine activation of nAChRs influences glutamate production, which leads to increased glutamate neurotransmission [29,30], which in turn contribute to behavioral changes such as drug-seeking behavior. Another important factor is the vesicular glutamate transporter (VGLUT1), which is essential for the transport and release of glutamate from presynaptic glutamatergic neurons into the synaptic cleft; its expression has been linked to extracellular glutamate concentrations in the mesocorticolimbic pathways. 

A second key neurotransmitter is GABA, which attenuates the behavioral changes produced by neuron overexcitation. Short-term exposure to nicotine has been found to elevate GABA neurotransmission, while prolonged exposure is associated with desensitization to the GABAergic system [31]. This biphasic effect is also mediated by nicotine activation of nAChRs, with an initial response and subsequent desensitization after prolonged exposure [31,32]. The decline in GABA concentrations in the frontal cortex and increase in glutamate concentrations in the striatum were observed in mice exposed to e-cigarette vapors containing nicotine for six months [27]. The transport and release of GABA from presynaptic GABAergic neurons into the synaptic cleft depends on the vesicular GABA transporter (VGAT), the expression of which has been linked to extracellular GABA concentrations in the mesocorticolimbic pathways. 

Cigarette smoke exposure also has multiple effects on immune and inflammatory processes. For instance, it directly modulates nuclear factor-kappa B (NF-*k*B) [33,34]. It also inhibits the activity of histone deacetylase enzymes, which regulate gene expression through histone modification; this ultimately prevents binding and activity of DNA transcription factors [33]. Nicotine-containing e-cigarette vapors also interacts with the immune system by influencing levels of circulating cytokines, including the inflammatory cytokines leukemia inhibitory factor, angiopoietin 1, and matrix metalloprotease-3 [35]. Furthermore, maternal exposure to e-cigarettes containing nicotine has been found to modulate the levels of interleukin-4 (IL-4) and interferon-gamma (IFNγ) in offspring [36].

In this study, we investigated the effect of four-week exposure to e-cigarette aerosols containing nicotine on neurobehaviors such as recognition and short-term memory, and locomotor activity in mice. We aimed to establish a link between neurobehavioral alterations and molecular alterations in the hippocampus, specifically of VGAT and VGLUT1. We also investigated the neuroinflammatory profile associated with the consumption of nicotine via e-cigarettes.

## 2. Materials and Methods

### 2.1. Reagents and Chemicals

Primary antibodies against VGLUT1, VGAT, and PSD-95 were obtained from Synaptic System Company (Gottingen, Germany). Chemiluminescent substrates for protein quantification by Western blotting were procured from Thermofisher. (-)-Nicotine hydrogen tartrate salt was purchased from Sigma Aldrich (SML1236-1G). A solution of 70/30 vegetable glycerin (VG)/propylene glycol (PG) was prepared in the laboratory as the vehicle for use in e-cigarettes; this mixture is used in commercial e-cigarette products. VG, PG and nicotine mixture was prepared as performed in our previous chronic exposure work [23,27,37]. Moreover, we have used gas chromatography to confirm that nicotine in our prepared solutions compared to positive control.

### 2.2. Experimental Model

Male and female C57BL/6J wild-type mice were purchased from Jackson Laboratories. The mice were housed in 40 to 60% humidity at a room temperature of 22 ± 2 °C, fed with standard rodent chow, and provided with water *ad libitum*. All experimental procedures were approved in accordance with the guidelines of the institutional Animal Care and Use Committee (IACUC) and the Research Ethics Committee (REC) at King Saud University (ethics reference number SE-19-130). The rationale for using C57BL/6J mice was that chronic exposure to e-cigarettes has been found to modulate levels of excitatory and inhibitory neurotransmitters in the FC and striatum of these mice [27]. 

### 2.3. E-Cigarette and Exposure

For all animal exposure experiments, CSM-STEP smoking machine (CH Technologies, Westwood, NJ, USA) supplied with a pump, pump base, fittings, tubing, wetted surfaces, seal holder and holder base was applied. In our work, we used CORESTA protocol, which is recommended for E-cigarette studies. This protocol mentions that the puff period is 3 s and the puff interval is 30 s with puff volume of 55 mL.

Male (M) and female (F) C57BL/6J mice aging 5–8 weeks were divided into three groups of 8 individuals (4M and 4F) as follows: Air Control group: mice that inhaled only normal air during the four-week study period. The mice were daily exposed to e-cigarette vapors in the mornings starting at 7:00 am. Electronic cigarette vehicle group (E-cig Vehicle group): mice that inhaled vapors containing 70/30 VG/PG vehicle and a berry flavoring agent without nicotine throughout the study period. Electronic cigarette nicotine group (E-cig Nicotine group): mice that inhaled vapors containing nicotine (25 mg/mL), a 70/30 VG/PG vehicle, and a berry flavoring throughout the study period. This dose is approximately similar to that used in our previous studies [23,27]. All mice were placed in whole-body exposure chambers (cages) inside a hood, supplied with a ventilation system, and exposed to the treatment for 3 s every 30 s, for one hour per day, for five days per week over four weeks, as previously performed [38].

At the end of the experiment, behavioral studies were conducted, following which the mice were anesthetized using a combination of 50 mg/mL ketamine and 20 mg/mL xylazine. Brains of the mice were then collected and stored at −80 °C for molecular studies. 

### 2.4. Behavioral Studies

Mouse memory function and locomotion were evaluated using long-term memory and locomotor assays, respectively. These behavioral assays were performed after the last exposure. For the long term memory assay, one day before last day of exposure, mice were first placed for 10 min in a specific chamber containing two identical objects. On the following day, one of the objects was replaced with a non-similar object and each mouse was once again placed in the chamber. Memory function was assessed in terms of the number of entries at each object using ANY-maze software (version 6). For the locomotion assay, animals were allocated 10 min for habituation and 10 min for testing, and the distance each mouse traveled during the test period was determined using activity monitoring software.

The Y-maze assay was used to investigate the degree to which the mice were willing to explore new environments. The alterations were calculated by measuring the number of times that mice alternate the three different arms consecutively. The percentage of spontaneous alteration was determined using the following equation:

(1) $$\%\;\mathrm{Spontaneous}\;\mathrm{alteration}\;\left(\%\mathrm{SAP}\right)=\left[\frac{\mathrm{Number}\;\mathrm{of}\;\mathrm{alterations}}{\mathrm{Total}\;\mathrm{arm}\;\mathrm{entries}-2}\right]\times 100$$

### 2.5. Brain Harvesting

At the end of the study, the brains of the mice were extracted, flash-frozen in liquid nitrogen, and stored at −80 °C for Western blotting. The hippocampus (HIP; ~2 mm of thickness) was then identified and located using a mouse brain atlas [39]. 

### 2.6. Western Blotting

Western blots were prepared as previously described in [23]. In brief, brain tissues of the HIP (n = 5–6 mice per group) were homogenized in RIPA lysis buffer containing phosphatase and protease inhibitors. Total protein was quantified using a NanoDrop Spectrophotometer, following which equal amounts were loaded on 8–15% sodium dodecyl sulphate-polyacrylamide gels for electrophoresis (SDS-PAGE). Separated proteins were transferred to polyvinylidene difluoride (PVDF) membranes using a transfer chamber. Blocking of membranes was then performed with 3% non-fat milk in 1 × tris buffered saline with tween (TBST) for 1 h followed by incubation with primary antibodies. The primary antibodies include rabbit anti-VGLUT1 polyclonal antibody (Synaptic System, Goettingen, Lower Saxony, Germany), rabbit anti-VGAT polyclonal antibody (Synaptic System, Goettingen, Lower Saxony, Germany), mouse anti-PSD-95 monoclonal antibody (Synaptic System, Goettingen, Lower Saxony, Germany) mouse anti-actin antibody. On the following day, the PVDF membranes were incubated with secondary antibodies in 3% non-fat milk in 1 × TBST for 90 min. Finally, Western blotting detection reagents were added for signal detection, which was conducted using an imaging system (ChemiDocTMMP-Bio-Rad). Image J software (Java 8) was then used for protein expression determination. 

### 2.7. Multiplex Cytokine Assay

To identify brain cytokine contents, a brain hemisphere was lysed using a lysis buffer-containing protease and phosphatase inhibitors. The amount of proteins in each lysate was quantified using a NanoDrop Spectrophotometer. Equal amount of proteins from each sample were analyzed using Luminex technology, an inflammatory-focused mouse cytokine and chemokine array assay. This technology utilizes bead-based technology to quantify target proteins. Of particular interest were 6Cklne/Exodus 2, Fractalkine, IFNβ-1, IL-16, MCP-5, and TIMP-1. All data were normalized to the average of the air-control group values in each protein.

### 2.8. Statistical Analysis

Statistical analysis was performed using GraphPad Prism^®^ software (version 6). One-way analysis of variance (ANOVA) followed by a Tukey’s post hoc test was used to identify differences between males and females in weekly average body weight during the study. The same combination of analyses was also conducted to determine any significant differences in behavioral and molecular data between groups. A value of *p* < 0.05 was considered significant. Unpaired *t*-tests were conducted to compare brain inflammatory markers between the E-cig Nicotine group and the Air Control group, and also to compare behavioral parameters between female and male mice in the E-cig Nicotine group. *T*-test was used to compare between number of enteries into the same object zone and number of enteries into the novel object zone within each group. 

## 3. Results

### 3.1. Effects of E-Cigarette Aerosols Containing Nicotine on Average Body Weight

**Figure 2:**
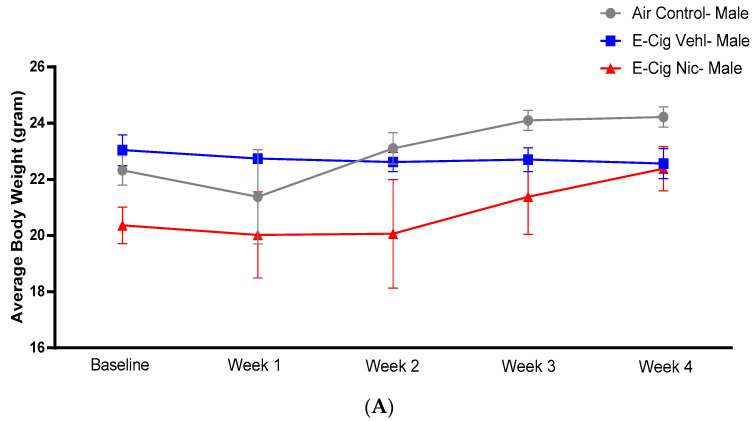

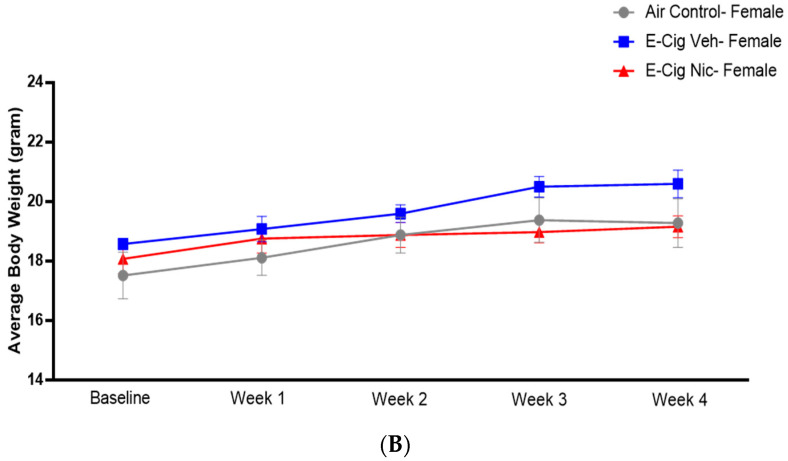
Average body weight of (**A**) male and (**B**) female mice of the three groups (Air control, E-Cig Veh, E-Cig Nic) during the study (four weeks). One-way analysis of variance (ANOVA) did not show any significant changes between the three groups in female and male mice. Data are presented as mean ± SEM (n = 5/group). E-Cig; electronic cigarettes, E-Cig Veh; electronic cigarette vehicle, E-Cig Nic; electronic cigarette nicotine.

To determine whether e-cigarette aerosols containing nicotine affected body weight, we measured the average weekly body weight. A two-way ANOVA followed by Tukey’s post hoc analysis did not identify any significant differences in the average body weight of male mice (Figure 2A) nor in that of female mice (Figure 2B). 

### 3.2. Effects of E-Cigarette Aerosols Containing Nicotine on Object Recognition Memory

#### 3.2.1. Same Object Recognition Memory

**Figure 3:**
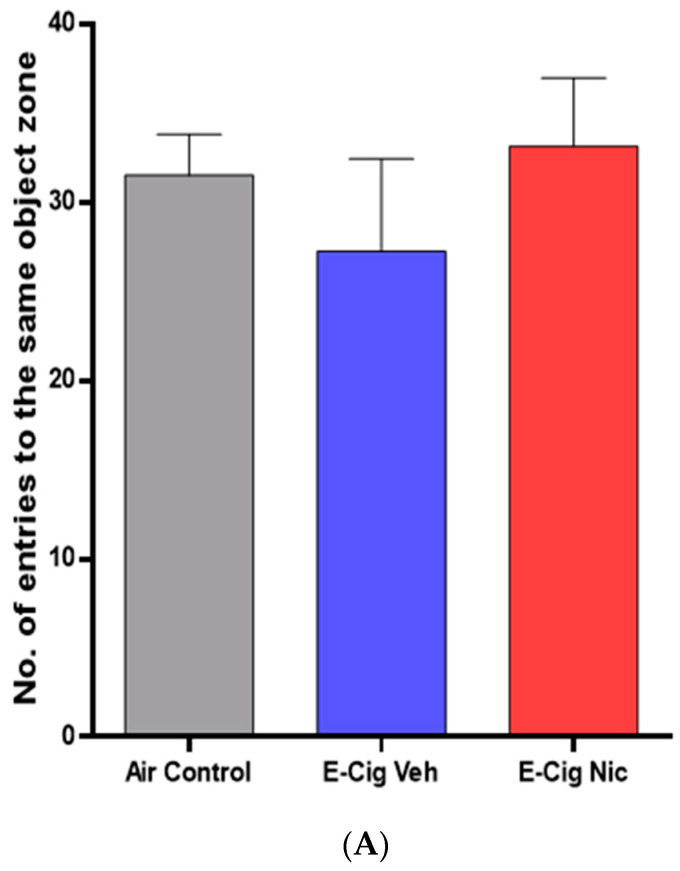

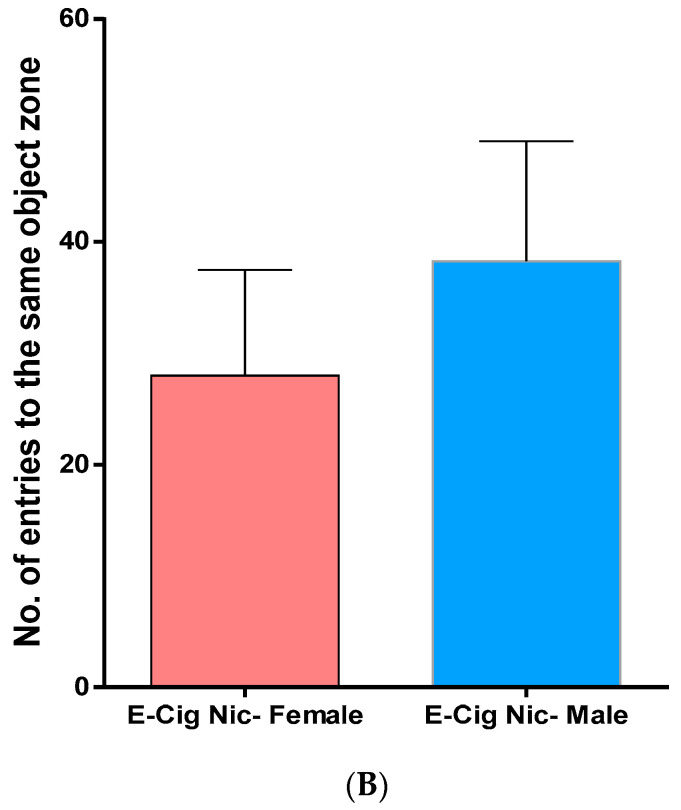
Recognition memory behaviors following four-week inhalation of e-cigarette vapors containing nicotine. (**A**) No. of entries into the same object zone (n = 8/group, 4F and 4M). One-way analysis of variance (ANOVA) did not show significant differences between the three groups. (**B**) No. of entries into the same object zone between male and female mice in the E-cig Nic group (n = 4/group). Unpaired *t* test also did not reveal any significant changes in the same zone number entries between male and female of E-cig Nic group. Data are presented as mean ± SEM. E-cig, electronic cigarette; E-cig Veh, electronic cigarette with vehicle only; E-cig Nic, electronic cigarette with nicotine; F, female; M, male.

We also investigated the effects of e-cigarette aerosols containing nicotine on same object recognition memory. We did not observe any between-group differences in the number of entries into the same object zone (Figure 3A), nor any differences between males and females in the E-cig Nicotine group (Figure 3B). 

#### 3.2.2. Long-Term Memory Test

**Figure 4:**
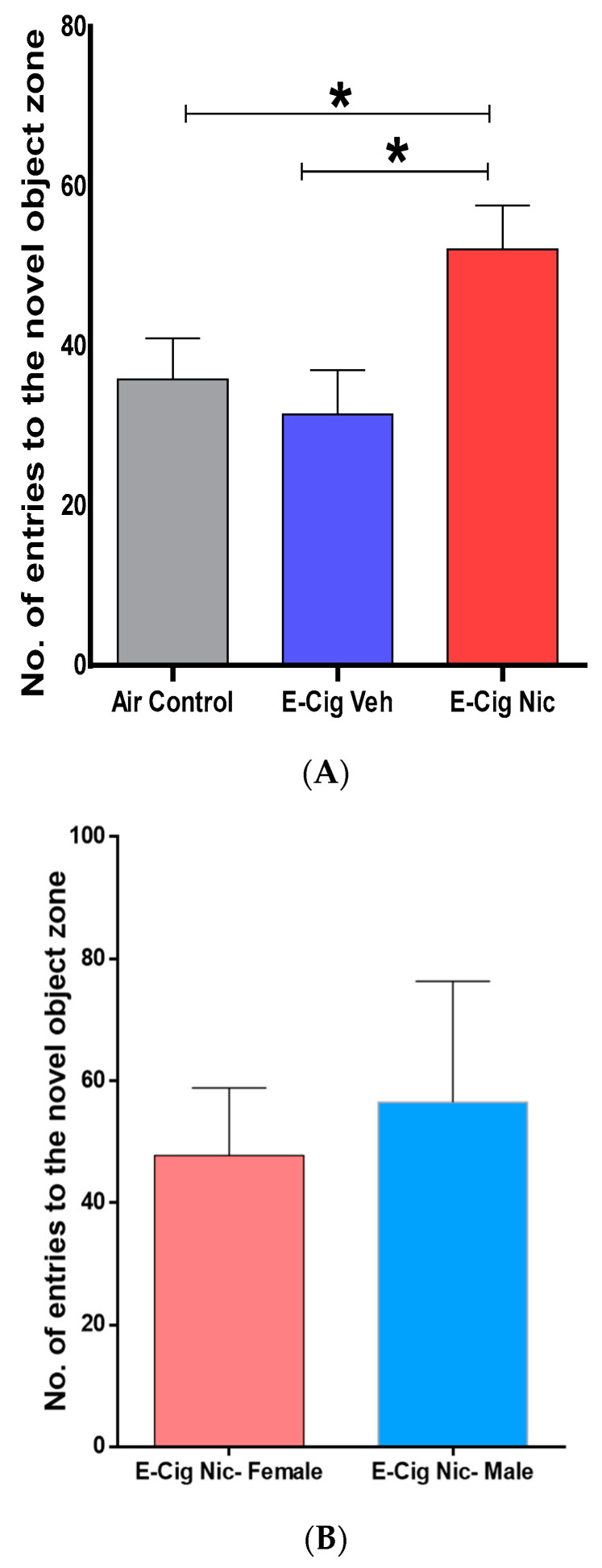

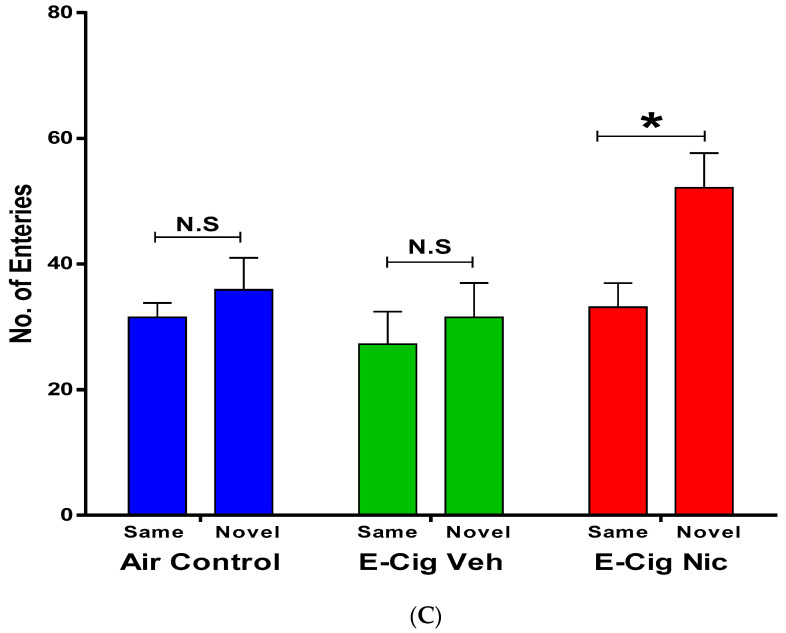
Recognition memory behaviors following four-week inhalation of e-cigarette vapors containing nicotine. (**A**) No. of entries into the novel object zone (n = 8/group, 4F and 4M). One-way analysis of variance (ANOVA) followed by a Tukey’s post hoc showed a significant increase in the novel object zone entries in E-cig Nic group as compared to controls. (**B**) No. of entries into the novel object zone between male and female mice in the E-cig Nic group (n = 4/group). Unpaired *t*-test also did not reveal any significant changes in the novel zone number entries between male and female of E-cig Nic group. (**C**) *t*-test showed that the number of entries into the novel object zone was significantly higher than number of entries into the same object zone in E-cig Nic group. This effect was not observed in either air or vehicle control groups (n = 8/group, 4F and 4M). Data are presented as mean ± SEM. (* *p* < 0.05). E-cig, electronic cigarette; E-cig Veh, electronic cigarette with vehicle only; E-cig Nic, electronic cigarette with nicotine; N.S, not significant; F, female; M, male.

We performed the long-term memory test to evaluate recognition memory behaviors following four-week inhalation of e-cigarette aerosols containing nicotine. A one-way ANOVA followed by Tukey’s post hoc analysis revealed that mice exposed to aerosols containing nicotine performed a significantly greater number of entries into the novel object zone compared with the Air Control and E-cigarette Vehicle groups (Figure 4A). Within the E-cigarette Nicotine group, no differences were observed between females and males in the number of entries into the novel object zone (Figure 4B). Statistical analysis revealed that the number of entries into the novel object zone was significantly higher than number of entries into the same object zone in E-cig Nic group (Figure 4C). This effect was not observed in either air or vehicle control groups (Figure 4C).

### 3.3. Effects of E-Cigarette Aerosols Containing Nicotine on Locomotion-Related Behaviors

**Figure 5:**
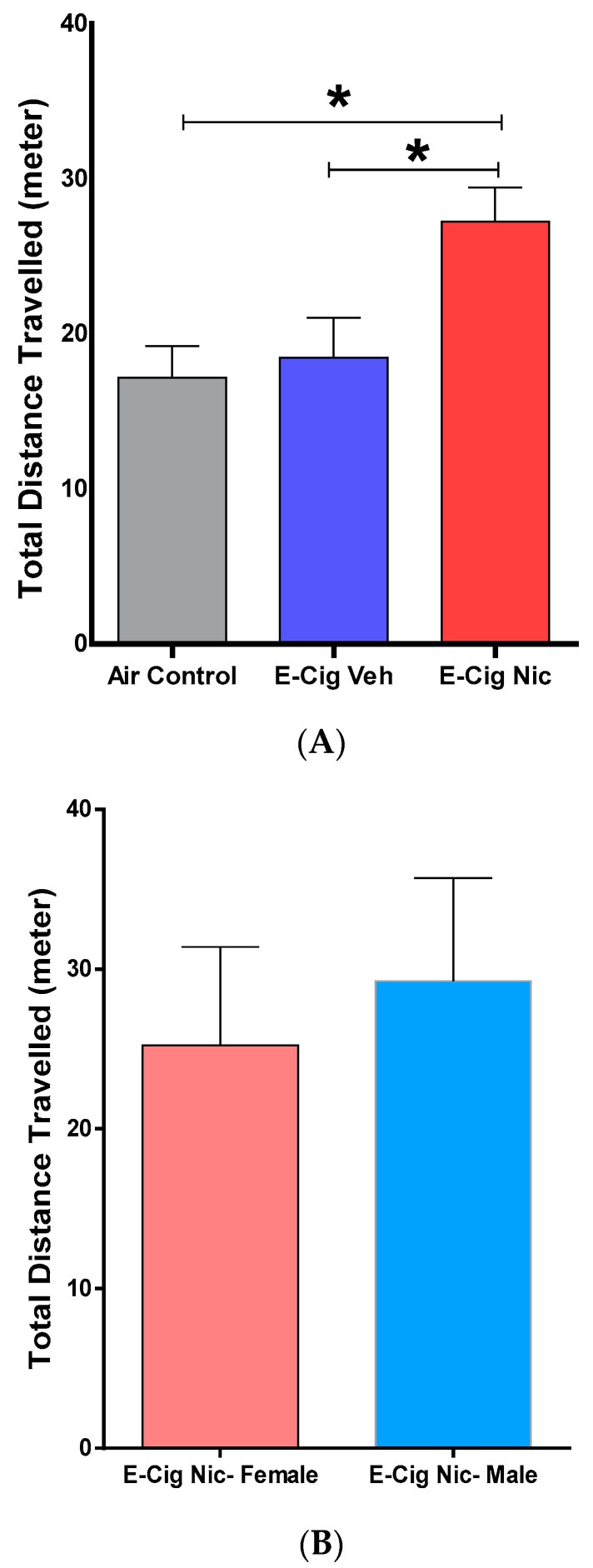
Locomotion-related behaviors following four-week inhalation of e-cigarette vapors containing nicotine. (**A**) Total distance traveled for each group (n = 8/group, 4F and 4M). One-way analysis of variance (ANOVA) followed by a Tukey’s post hoc showed a significant increase in the total distance traveled in E-cig Nic group as compared to controls. (**B**) Total distance traveled for female and male mice in the E-cig Nic group (n = 4/group). (* *p* < 0.05). Unpaired *t* test also did not reveal any significant changes in the total distance traveled between male and female of E-cig Nic group. Data are presented as mean ± SEM. E-cig, electronic cigarette; E-cig Veh, electronic cigarette with vehicle only; E-cig Nic, electronic cigarette with nicotine; F, female; M, male; F, female; M, male.

We then determined the effects of e-cigarette aerosols containing nicotine on locomotion-related behaviors using locomotor tests. A one-way ANOVA followed by Tukey’s post hoc analysis revealed that mice exposed to aerosols containing nicotine traveled a significantly greater total distance compared with the Air Control and E-cigarette Vehicle groups (Figure 5A). Within the E-cigarette Nicotine group, an unpaired *t*-test did not reveal any significant difference between male and female mice in total distance traveled (Figure 5B). 

### 3.4. Effects of E-Cigarette Aerosols Containing Nicotine on Spatial Memory

**Figure 6:**
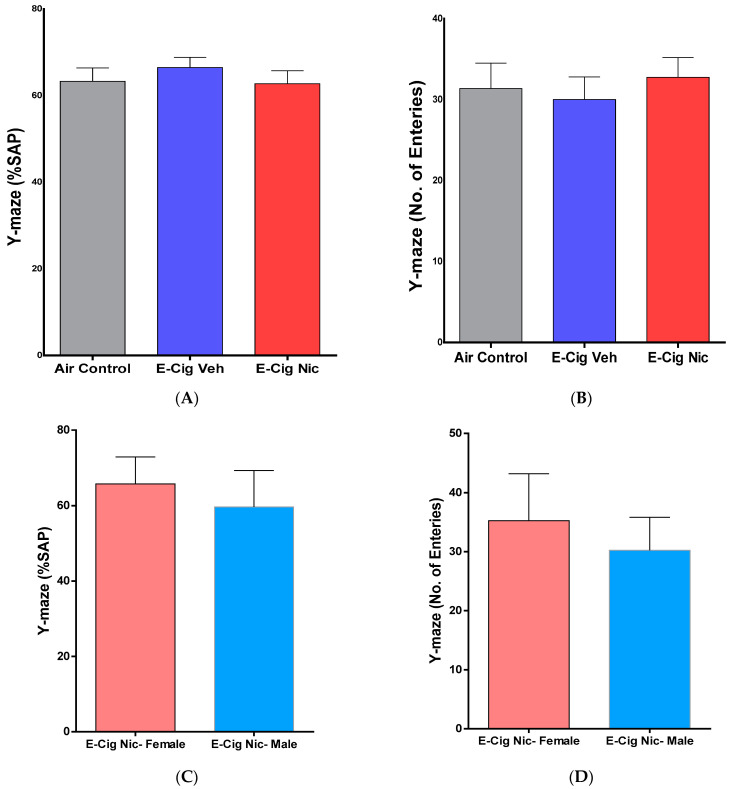
Spatial memory behaviors following four-week inhalation of e-cigarette vapors containing nicotine. (**A**) Percentage of spontaneous alterations (%SAP) (n = 8/group, 4F and 4M). (**B**) Number of entries into Y-maze arms (n = 8/group, 4F and 4M). (**C**) %SAP in female and male mice of the E-cig Nic group (n = 4/group). (**D**) Number of entries into Y-maze arms in female and male mice of the E-cig Nic group (n = 4/group). One-way analysis of variance (ANOVA) did not show significant differences between the three groups. Unpaired *t* test also did not reveal any significant changes in the Y maze parameters (%SAP and number of entries) between male and female of E-cig Nic group. Data are presented as mean ± SEM. E-cig, electronic cigarette; E-cig Veh, electronic cigarette with vehicle only; E-cig Nic, electronic cigarette with nicotine; F, female; M, male.

We also investigated the effects of e-cigarette vapors containing nicotine on spatial memory using the Y-maze test. A one-way ANOVA followed by Tukey’s post hoc analysis revealed no difference between the three groups in the percentage of spontaneous alterations to explore new areas of the Y-maze equipment (Figure 6A). Similarly, no difference was observed in the number of entries into Y arms (Figure 6B). Additionally, unpaired *t*-tests comparing male and female mice on these parameters did not identify any significant differences (Figure 6C,D). 

### 3.5. Effects of E-Cigarette Aerosols Containing Nicotine on VGLUT1 Expression in the Hippocampus

**Figure 7:**
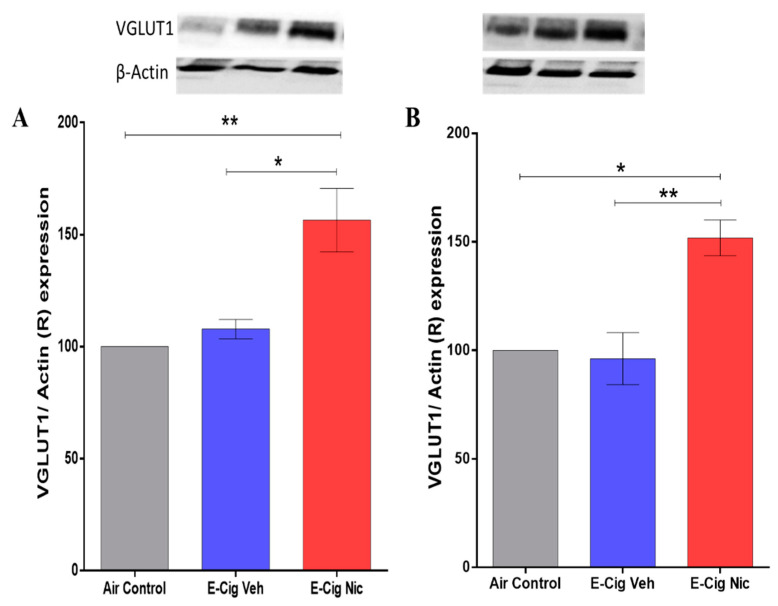
Relative (R) expression of VGLUT1 in the hippocampus following four-week inhalation of e-cigarette vapors containing nicotine. (**A**) Representative VGLUT1 expression blots in the hippocampus of males of all three groups (upper panel) and corresponding quantitative statistical analysis (lower panel). (**B**) Representative VGLUT1 expression blots in the hippocampus of females of all three groups (upper panel) and corresponding statistical analysis (lower panel). One-way analysis of variance (ANOVA) followed by a Tukey’s post hoc showed a significant increase in the the expression of VGLUT1 in the hippocampus in E-cig Nic group as compared to controls in both male and female mice. Data are presented as mean ± SEM (n = 3/group). E-cig, electronic cigarette; E-cig Veh, electronic cigarette with vehicle only; E-cig Nic, electronic cigarette with nicotine. (* *p* < 0.05, ** *p* < 0.01).

We determined the effects of e-cigarette aerosols containing nicotine on VGLUT1 expression in the hippocampus using Western blotting. A one-way ANOVA followed by Tukey’s post hoc analysis revealed that mice exposed to aerosols containing nicotine exhibited significantly higher expression of VGLUT1 in the hippocampus compared with the Air Control and E-cigarette Vehicle groups (Figure 7).

### 3.6. Effects of E-Cigarette Aerosols Containing Nicotine on VGAT Expression in the Hippocampus

**Figure 8:**
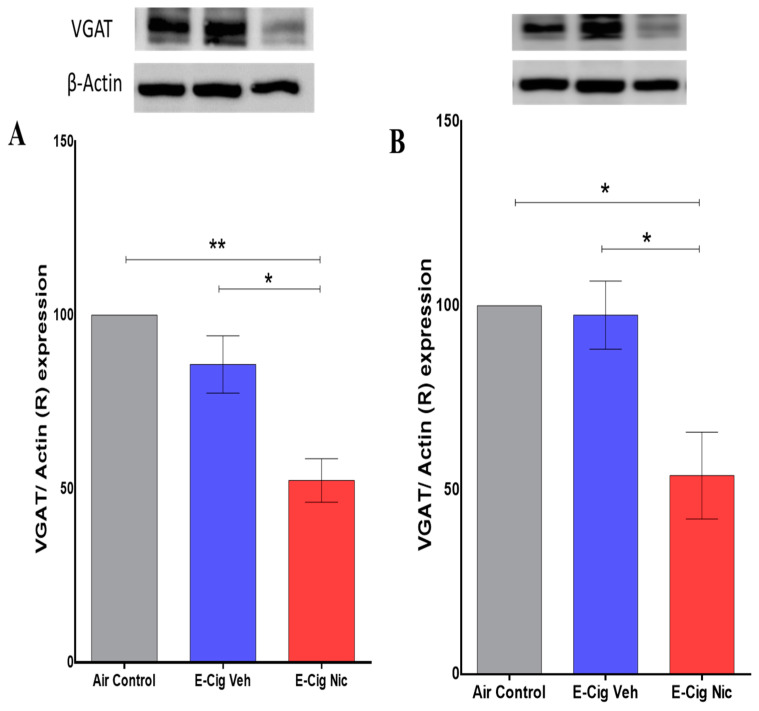
Relative (R) expression of VGAT in the hippocampus following four-week inhalation of e-cigarette vapors containing nicotine. (**A**) Representative VGAT expression blots in males of all three groups (upper panel) and corresponding quantitative statistical analysis (lower panel). (**B**) Representative VGAT expression blots in females of all three groups (upper panel) and corresponding statistical analysis (lower panel). One-way analysis of variance (ANOVA) followed by a Tukey’s post hoc showed a significant increase in the the expression of VGAT in the hippocampus in E-cig Nic group as compared to controls in both male and female mice. Data are presented as mean ± SEM (n = 3/group). E-cig, electronic cigarette; E-cig Veh, electronic cigarette with vehicle only; E-cig Nic, electronic cigarette with nicotine. (* *p* < 0.05, ** *p* < 0.01).

Also using Western blot, we further quantified expression of VGAT in the hippocampus after inhalation of e-cigarette aerosols containing nicotine. A one-way ANOVA followed by Tukey’s post hoc analysis revealed that mice exposed to aerosols containing nicotine exhibited significantly lower expression of VGAT in the hippocampus compared with the Air Control and E-cigarette Vehicle groups (Figure 8). 

### 3.7. Effects of E-Cigarette Aerosols Containing Nicotine on PSD-95 Expression in the Hippocampus

**Figure 9:**
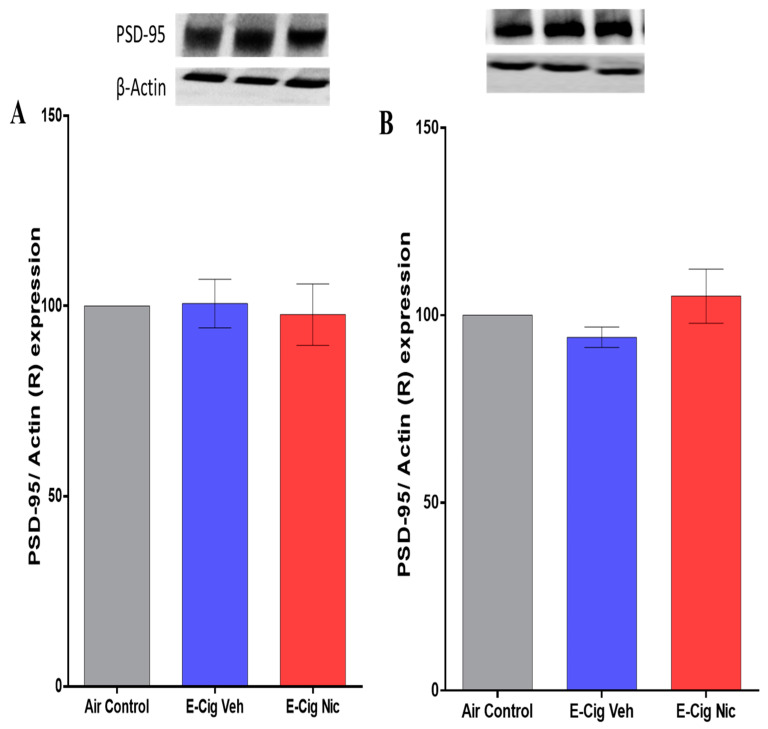
Relative (R) expression of PSD-95 in the hippocampus following four-week inhalation of e-cigarette vapors containing nicotine. (**A**) Representative PSD-95 expression blots in males of all three groups (upper panel) and corresponding quantitative statistical analysis (lower panel). (**B**) Representative PSD-95 expression blots in females of all three groups (upper panel) and corresponding statistical analysis (lower panel). One-way analysis of variance (ANOVA) did not show significant differences between the three groups in the hippocampal expression of the PSD-95. Data are presented as mean ± SEM (n = 3/group). E-cig, electronic cigarette; E-cig Veh, electronic cigarette with vehicle only; E-cig Nic, electronic cigarette with nicotine.

Western blot was then used to determine the expression of PSD-95 in the hippocampus after inhalation of e-cigarette aerosols containing nicotine. A one-way ANOVA followed by Tukey’s post hoc analysis revealed no significant between-group differences in PSD-95 expression (Figure 9).

### 3.8. Effects of E-Cigarette Aerosols Containing Nicotine on Neuro-Inflammatory Cytokines and Chemokines

**Figure 10:**
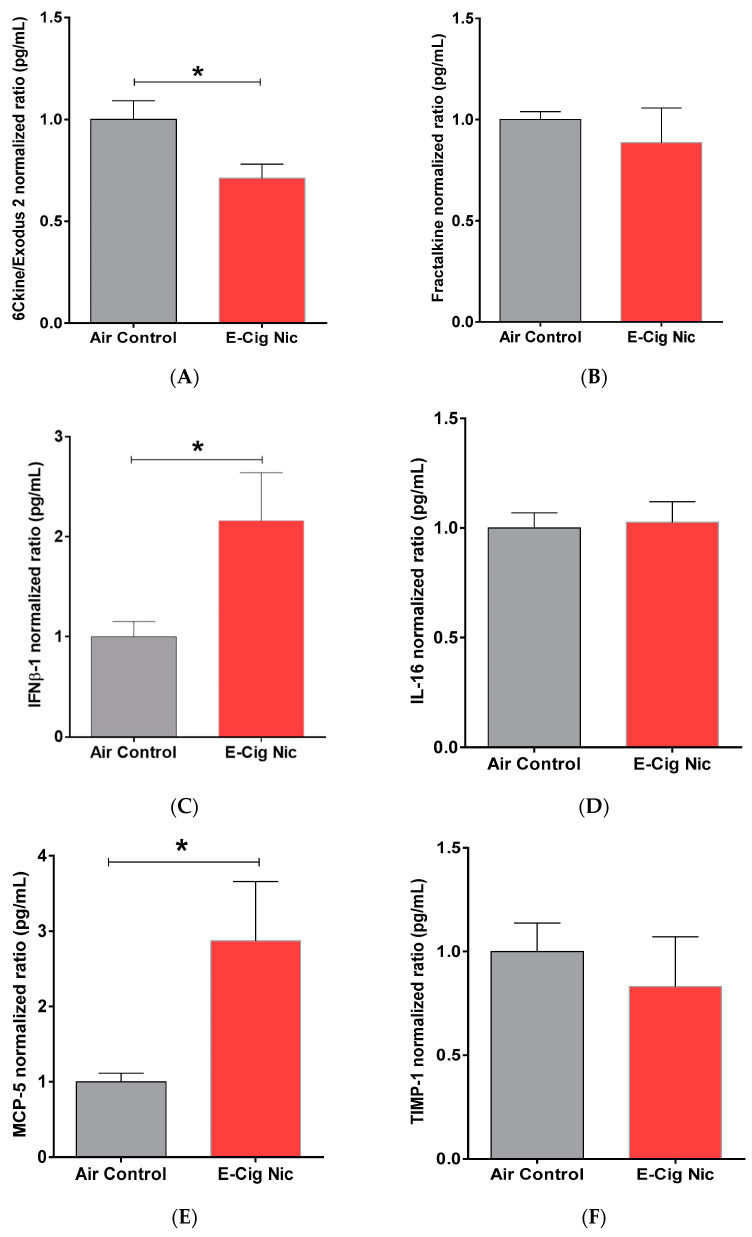
Brain concentration of neuro-inflammatory cytokines and chemokines in the Air Control and E-cig Nicotine groups after four-week inhalation of e-cigarette aerosols. (**A**) 6Cklne/Exodus 2. (**B**) Fractalkine. (**C**) IFNβ-1. (**D**) IL-16. (**E**) MCP-5. (**F**) TIMP-1. N = 4–5/biological replicates (M or F = 2–3). and 8–10/technical replicates. Unpaired *t* test was used to compare each marker between air control and E-cig Nic groups. Data are presented as mean ± SEM. (* *p* < 0.05). E-cig, electronic cigarette; E-cig Nic, electronic cigarette with nicotine; F, female; M, male.

Since we found significant alterations in neurobehavioral parameters as well as VGLUT1 and VGAT expression between air control and E-cig Nicotine groups, we measured the protein content of neuro-inflammatory chemokines in the entire brain after inhalation of e-cigarette aerosols containing nicotine (Figure 10). We found that the E-cig Nicotine group displayed significantly less chemokine (C-C motif) ligand 21 (6Cklne/Exodus 2) compared with the Air Control group (Figure 10A). In addition, the E-cig Nicotine group exhibited significantly increased levels of interferon beta-1 (IFNβ-1) (Figure 10C) and monocyte chemotactic protein (MCP-5) (Figure 10E) relative to the Air Control group. We did not observe any significant between-group differences in brain content of chemokine (C-X3-C motif) ligand 1 (Fractalkine) (Figure 10B), interleukin-16 (IL-16) (Figure 10D), or metallopeptidase inhibitor 1 (TIMP-1) (Figure 10F).

## 4. Discussion

Our study revealed that mice treated with e-cigarette vapors containing nicotine experience a significant enhancement of memory capabilities, as measured by long-term memory testing. This finding is supported by previously published studies that have demonstrated the effects of nicotine exposure on memory behaviors [40,41,42,43]. Specifically, memory cognition and locomotion behaviors were increased in animals chronically exposed to nicotine relative to the control group. We did not observe significant differences between male and female mice in terms of behavioral parameters (n = 4/gender); however, studies with larger sample sizes are required to confirm these conclusions. A previous study found that chronic intermittent exposure to e-cigarette vapors-containing nicotine was associated with increased locomotor activity in *ApoE*−/− mice [44] and several-week exposure to nicotine attenuated methamphetamine-dysregulated novel object recognition [41]. 

At the molecular level, we found that mice exposed to nicotine-containing aerosols exhibited increased hippocampal expression of VGLUT1, an essential transporter of glutamate into the synaptic cleft in the mesocorticolimbic system. In addition, this elevated expression may result in increased glutamate release. These results align with those of a previous study that revealed a significant increase in glutamate concentration in the striatum of C57BL/6J mice exposed to nicotine-containing vapors for six months [27]. It is important to note that glutamatergic projections from the hippocampus to the nucleus accumbens (a ventral striatum major component) was documented previously [45].

Previous studies have reported that exposure to e-cigarette vapors containing nicotine chronically reduces the expression of astroglial glutamate transporters, including glutamate transporter-1 (GLT-1) in the striatum and hippocampus, and cystine/glutamate antiporter (xCT) in the hippocampus [23,37]. They documented lower expression of GLT-1 and xCT in animals exposed for three and six months. Another study revealed that three-month exposure to vapors containing 50 mg/mL nicotine modulated levels of metabotropic glutamate receptors-1 (mGluR1) and mGluR5 expression in the nucleus accumbens shell [28]. In conjunction with this prior data, our findings suggest that as a consequence of reduced glutamate uptake, nicotine could increase the release of glutamate into the synapses in the mesocorticolimbic system.

We also found that chronic exposure to nicotine-containing aerosols decreases hippocampal expression of VGAT, an essential protein for GABA transport into the synapses. Furthermore, our results indicate that this decreased VGAT expression might reduce GABA concentrations. This is consistent with a previous study that revealed significantly decreased concentrations of GABA in the frontal cortex of C57BL/6J mice exposed to nicotine-containing vapors for six months [27]. Importantly, GABAergic projections are reported to be projected from the hippocampus and these projections innervate the frontal cortex [46]. Multiple studies similarly indicated that exposure to nicotine could lead to desensitization of the GABA system [31,32], possibly the presynaptic nicotinic receptors. More importantly, the rate of desensitization of nAChRs in glutamatergic neurons is slower than that of nAChRs in GABAergic neurons [31]. This reduces in the GABA-mediated inhibition of dopamine release, leading to greater excitability of the dopamine reward system, which in turn enhances reinforcement of nicotine self-administration [31]. Based on these findings, we suggest that chronic nicotine inhalation might exert a desensitizing effect on the GABAergic system. A short-term increase in the frequency of GABA postsynaptic currents is associated with continuous exposure to low nicotine concentrations, and may result from desensitization of presynaptic nAChRs [47]. In addition, chronic exposure to nicotine increases transcription of corticotropin-releasing hormone (Crh) mRNA, which simulates the corticotropin-releasing factor in dopaminergic neurons of the ventral tegmental area and consequently inhibits GABAergic transmission to dopaminergic neurons [48]. Conversely, use of a viral vector to decrease Crh mRNA expression in dopaminergic neurons has been found to prevent nicotine-induced GABA neurotransmitter dysregulation [48]. Thus, the evidence indicates that stimulating GABA release reduces dopamine-mediated nicotine dependence. 

It has been reported that exposure to 500 nM nicotine for 25 min elevates GABA release with subsequent long-term inhibition of GABAergic neurons, as evidenced by the frequency of spontaneous inhibitory postsynaptic currents. At the same time, the same exposure increases glutamatergic activity without any long-term inhibitory effect on glutamatergic neurons, as determined by spontaneous excitatory postsynaptic currents. These findings indicate that nicotine plays a role in GABAergic system desensitization [32].

In addition to the above neuronal effects, we found that chronic inhalation of nicotine-containing aerosols leads to a reduction in the brain protein content ratio of chemokine ligand 21 (CCL21), which can regulate the migration of dendritic cells to lymphoid cells [33]. We also observed increased interferon beta-1 (IFNβ-1) protein content ratio in the brain following nicotine exposure. A previous study reported that nicotine-induced increase in the IFNβ-1 may be attributable to the stimulation of NF-*k*B [49]. In addition, a study of daily exposure to nicotine vapors in C57BL/6 and CD-1 mice revealed increased interferon gamma (IFNγ) in the lungs [35]. Both of these findings support our results regarding increased interferon level. We also found that nicotine-exposed mice have higher MCP-5 levels in the brain than control mice. A previous clinical study similarly found that blood concentrations of chemo-attractant protein-1 (MCP-1) increase with smoking duration [50]. Altogether, our data provides critical information regarding the ability of nicotine-containing e-cigarette vapors to increase pro-inflammatory biomarkers in the brain. A previous study reported that one and three-month exposure to E-cigarette vapors-containing nicotine and flavors is associated with increased gene expression of neuroinflammatory cytokines such as tumor necrosis factor, IL-1β and IL-6 in the accumbens shell [38]. However, because we combined male and female mice samples, more studies are required to explore the role of sex differences in nicotine modulation of chemokine/cytokine levels. 

**Figure 11:**
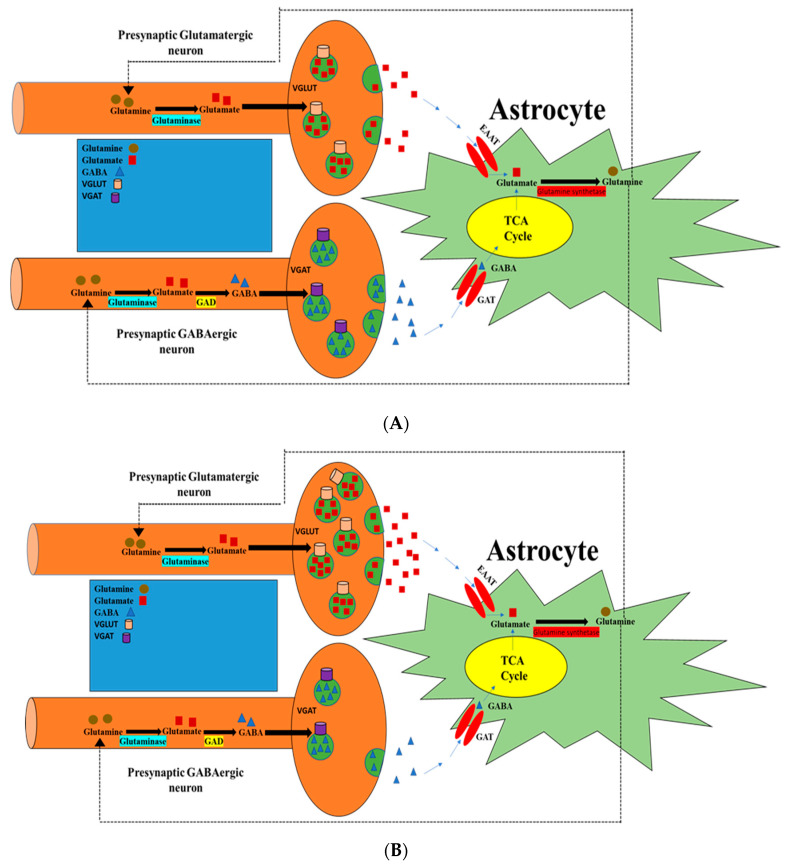
Schematic showing the effects of nicotine on expression of VGLUT1 and VGAT in presynaptic glutamatergic and GABAergic neurons, respectively. (**A**) Normal expression of VGLUT1 and VGAT. (**B**) E-cig aerosols containing nicotine modulate expression of VGLUT1 and VGAT and, consequently, the release of glutamate and GABA. This diagram does not include effects on different parameters identified in other studies. VGLUT, vesicular glutamate transporter; VGAT, vesicular GABA transporter; EAAT, excitatory amino acid transporter; GAD, glutamate decarboxylase; GAT, GABA transporter; TCA, tricarboxylic acid.

In conclusion, our study may suggest that that there are roles of nicotine-containing e-cigarette aerosols in modulating the hippocampal expression of VGLUT1 and VGAT. This modulatory effect might extend into glutamate and GABA homeostasis in the mesocorticolimbic system (Figure 11), and hence may explain the behavioral changes that were observed. Additional studies are needed to explore the effects of longer exposure on neurobehaviors, as greater duration may be required for the observed changes in VGAT and VGLUT1 expression and in glutamate and GABA homeostasis to negatively affect neurobehaviors. However, a larger sample size to confirm the role of sex differences in these markers, including behavioral and molecular markers, is important. In addition, to compare the chronic and withdrawal phases of nicotine exposure models, it is essential to investigate the respective roles of presynaptic and postsynaptic markers of excitatory and inhibitory neurotransmitters during the withdrawal phase after chronic exposure to e-cigarette vapors containing nicotine.

## Data Availability

Data are contained within the article.
